# Efficacy of probiotics on stress in healthy volunteers: A systematic review and meta‐analysis based on randomized controlled trials

**DOI:** 10.1002/brb3.1699

**Published:** 2020-07-14

**Authors:** Ning Zhang, Yanan Zhang, Menglin Li, Weiguang Wang, Zhenzhu Liu, Chongcheng Xi, Xunying Huang, Jintao Liu, Junwei Huang, Dong Tian, Jie Mu, Xing Liao, Shuangqing Zhai

**Affiliations:** ^1^ School of Traditional Chinese Medicine Beijing University of Chinese Medicine Beijing China; ^2^ Center for Evidence Based Chinese Medicine Institute of Basic Research in Clinical Medicine China Academy of Chinese Medical Sciences Beijing China; ^3^ School of Integrated Traditional Chinese and Western Medicine Hebei University of Chinese Medicine Hebei China; ^4^ Department of Traditional Chinese Medicine Beijing Hospital Beijing China

**Keywords:** gastrointestinal microbiome, healthy volunteers, meta‐analysis, probiotics, psychological stress, systematic review

## Abstract

**Background:**

Probiotics seems to play a beneficial role in stressed populations; thus, a systematic review and meta‐analysis to assess the effects of probiotics on stress in healthy subjects were conducted.

**Methods:**

Randomized controlled trials on the effects of probiotics on stress in healthy subjects were retrieved from five databases. The effects of probiotics on subjective stress level, stress‐related subthreshold anxiety/depression level, cortisol level, and adverse reactions were analyzed. Separate subgroup analyses were conducted on single‐strain probiotics versus multi‐strain probiotics, and short‐term administration versus long‐term administration.

**Results:**

Seven studies were included, involving a total of 1,146 subjects. All the studies were rated as low or moderate risk of bias. Our research found that probiotic administration can generally reduce the subjective stress level of healthy volunteers and may improve their stress‐related subthreshold anxiety/depression level, but no significant effect was observed in the subgroup analysis. The effect of probiotics on cortisol level was not significant. Adverse reactions were reported in only one of seven studies, but left undescribed.

**Conclusion:**

Current evidence suggests that probiotics can reduce subjective stress level in healthy volunteers and may alleviate stress‐related subthreshold anxiety/depression level, without significant effect on cortisol level, and there is not enough support to draw conclusions about adverse effects; thus, more reliable evidence from clinical trials is needed.

## Graphical abstract

This is a systematic review and meta‐analysis based on randomized controlled trials to assess the effects of probiotics on stress in healthy volunteers.

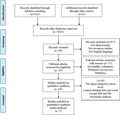

## 1 INTRODUCTION

Stress is a challenging and threatening experience in psychology and physiology, involving individual and environmental factors, historical events and current stress experiences, and interactions between psychological and physiological reactivity (Epel et al., 2018; Jamieson, Mendes, & Nock, 2013). When an individual perceives that environmental demands exceed its adaptive capacity, stress occurs (McEwen, 2007). Stress is ubiquitous in daily life and affects us all the time. The stress we can control is so called “positive stress,” which allows us to adapt constantly changing environment with excitement and accomplishment (Jamieson et al., 2013). This process is achieved by causing many transient physiological/psychological reactions such as tension (Holte, Vasseljen, & Westgaard, 2003), anxiety (Schneiderman, Ironson, & Siegel, 2005), elevated blood pressure (Lambiase, Dorn, & Roemmich, 2013), and increased heart rate (Rimmele et al., 2007). When we suffer from some high‐level, long‐term, uncontrolled “negative stress,” it will have a negative impact on physical and mental health and daily life. Studies have shown that excessive stress not only leads to irritability and hostility in emotions affecting social communications (Everson‐Rose et al., 2014; Vella & Friedman, 2009), and unhealthy lifestyles of smoking and alcohol abuse (Becker, 2017; Siegel, Korbman, & Erblich, 2017), but also increases the risk of hypertension (Liu, Li, Li, & Khan, 2017), cardiovascular diseases (Roemmich, Lambiase, Balantekin, Feda, & Dorn, 2014), digestive diseases (Choung & Talley, 2008; Pigrau et al., 2016), and mental disorders including depression and anxiety (Bekhbat & Neigh, 2018; Gehrman, Harb, Cook, Barilla, & Ross, 2015; Slavich & Irwin, 2014). According to the UK Health and Safety Executive Committee report (Jackson, 2014), occupational stress increased by about 30% throughout the UK from 1990 to 1995. Till 2009, 13.5 million working days were lost yearly due to stress. The annual economic costs associated with work stress were as high as £4.5 billion. Stress may come from all aspects of work, study, and social life (Brown, Richman, & Rospenda, 2017; Siegrist & Li, 2016; Zhang, Zhang, Zhang, Zhang, & Feng, 2018). There are some potential links with individual factors (Langgartner et al., 2017; Roy, Kirschbaum, & Steptoe, 2001; Tuvesson, Eklund, & Wann‐Hansson, 2012), and stress in daily life is often unavoidable. Therefore, exploring a simple, effective and feasible way to relieve stress in order to reduce the adverse effects of stress on health, work and life well‐being has become a hotspot in current research, especially in the medical field.

A large number of microorganisms are colonized in human guts, mainly including bacteria, archaea, protozoa, and viruses. They are symbiotic with the host and extensively participate in their multiple life activities (Bruce‐Keller, Salbaum, & Berthoud, 2018; Cryan, 2016; Heintz‐Buschart & Wilmes, 2018; Lloyd‐Price, Abu‐Ali, & Huttenhower, 2016). As research progresses, scientists have discovered that both genome of the human and gut microbiota are essential for maintaining health. Gut microbiota plays a crucial role in regulating physiological functions, including development and function of the central nervous system (O'Hara & Shanahan, 2006; Stilling, Dinan, & Cryan, 2014). With certain individual variations (Qin et al., 2010), gut microbiota is subject to dynamic changes due to gender, age, and lifestyle (Buford, 2017; Santoro et al., 2018; Valle Gottlieb, Closs, Junges, & Schwanke, 2018). In addition, psychological factors such as stress can induce complex changes in the intestinal flora, including community stability and species diversity (Dinan & Cryan, 2016; Marin et al., 2017). Studies have found that psychosocial stress can modify intestinal flora through certain bioactive factors (Bailey et al., 2011; Cryan & Dinan, 2012), and some of these factors, such as serotonin (Mittal et al., 2017), cortisol (Luo et al., 2018), and brain‐derived neurotrophic factor (Brzozowski et al., 2016), play important roles in the development of mental illness. Currently, some potential relations between gut microbiota and stress‐related diseases have been preliminarily confirmed (Liu, 2017), and the gut microbiota is more accessible and modifiable than the human genome in medicine, which also provides more chances for the prevention and treatment of stress‐related diseases by regulating the gut microbiota (Cenit, Sanz, & Codoner‐Franch, 2017; Dinan, Stanton, & Cryan, 2013).

Probiotics are active microbes that, when applied in sufficient amounts, can exert beneficial effects by regulating intestinal microecological balance (Gibson & Roberfroid, 1995). Probiotics are also known as “psychobiotics” because of their positive effects in emotion, cognition, and other psychological processes (Sarkar et al., 2016). In recent years, many studies have been carried out around the world that use probiotics to regulate psychiatric disorders. Studies found that under stress conditions, probiotics can play a beneficial role by regulating the synthesis and release of a variety of neurotransmitters and bioactive factors including cortisol (Takada et al., 2016), serum corticotropin‐releasing factor (CRF) (Yang et al., 2016), tumor necrosis factor‐α (TNF‐α) (Marcos et al., 2004), and to some extent improve the stress‐related physical and psychiatric symptoms of the subjects (Kato‐Kataoka et al., 2016; Langkamp‐Henken et al., 2015), which is expected to become a potential therapy or auxiliary measure for relieving stress. However, some studies have found that effects of probiotics on cognition and stress resilience in humans are scarce and sometimes contradictory, and there is currently no evidence‐based medical evidence of stress‐relieving effect for probiotics, so we conducted a systematic review and meta‐analysis for the data from all randomized controlled trials conducted in healthy subjects to date, focusing on whether probiotics alleviate the psychological/physiological stress of healthy subjects, and the possible adverse effects of probiotics, which are also important difference between our study and other systematic reviews and meta‐analysis of the potential effects of probiotics on mental illness (Fond et al., 2015; Huang, Wang, & Hu, 2016; Liu et al., 2018; Ng, Peters, Ho, Lim, & Yeo, 2018; Pirbaglou et al., 2016; Reis, Ilardi, & Punt, 2018; Wallace & Milev, 2017).

## 2 MATERIALS AND METHODS

Article search, trial selection, risk assessment of bias, and data extraction were completed by two authors (Yanan Zhang & Menglin Li). When there is a disagreement between the two authors, it will be resolved through discussion. If necessary, the third author (Ning Zhang) will arbitrate. The protocol of this study has been registered with the International Prospective Register of Systematic Reviews (PROSPERO) (ID: CRD42019122930), and the protocol has been published (Zhang et al., 2019).

### 2.1 Article search

The five databases of Cochrane Library, Embase, Medline (Ovid), PsycINFO (Ovid), and CINAHL (EBSCOhost) were searched from the earliest record to 23 March 2019 using the search terms “psychological stress,” “mental health,” “mental hygiene,” and “probiotics” (Zhang et al., 2019). References from these publications were also reviewed. For the retrieved research protocol, we further review the status, details, and publications indexed to this study at https://www.clinicaltrials.gov/ to ensure the comprehensiveness of the article search.

### 2.2 Inclusion and exclusion criteria

Eligible studies had to meet the following criteria: (a) described as a randomized controlled trial; (b) included participants were in a healthy state, without known major health problems; (c) the interventions were probiotic administration; (d) the comparisons should be placebo. In addition, the studies which could use probiotic alone in the experimental group compared with the control group were also deemed eligible; (e) published in English.

Studies with the following criteria were excluded from qualitative synthesis: (a) probiotics not survive; (b) the interventions were prebiotics; (c) not detailed or complete data reported in the research, and the author cannot provide relevant information; and (d) duplicate publications or secondary analysis of the same study.

### 2.3 Outcomes

#### 2.3.1 Primary outcomes

*Subjective stress level:* It is measured by the Perceived Stress Scale, Berocca Stress Index, Personal Strain Questionnaire of the Occupational Stress Inventory‐Revised Edition, or Visual Analog Scale, etc.

*Stress‐related subthreshold anxiety/depression level:* It is measured by the General Health Questionnaire, Psychological General Well‐Being Schedule, State/Energy Visual Analogue Scale, Hospital Anxiety and Depression Scale, Hamilton Anxiety Rating Scale, Depression Anxiety Stress Scale, Visual Analog Scale, Geriatric Depression Scale, or Hopkins Symptom Checklist‐90, etc.

#### 2.3.2 Secondary outcomes

*Cortisol level:* The cortisol level in saliva, plasma, serum, or urine.

*Adverse reactions:* Adverse reactions that may be associated with probiotic administration.

### 2.4 Data extraction

We extracted data from eligible studies using a pre‐experimental validation table. The content included the characteristics of the participants, the study area, the details of the interventions and controls, and data from all follow‐up points.

### 2.5 Risk of bias assessment

The Cochrane Collaboration's tool was used to assess the risk of bias in the included studies. The evaluation was conducted from the following seven aspects including the random sequence generation, allocation concealment, blinding of participants and personnel, blinding of outcome assessment, incomplete outcome data, selective reporting, and other bias (conflict of interest and registered protocol) and assessed as low risk, high risk, and unclear risk.

### 2.6 Statistical analysis

RevMan 5.3 software (RRID:SCR_00358, Cochrane) was used to quantify the included studies. For continuous variables (e.g., subjective stress level), the standardized mean difference (SMD) with 95% confidence intervals (95% CIs) was analyzed as summary statistics; for noncontinuous variables, qualitative analysis was only performed due to insufficient included researches. The Tau^2^, *I* ^2^, and chi‐square were calculated to assess statistical heterogeneity, and a fixed‐effect model was selected based on statistical heterogeneity. We also performed subgroup analyses based on the type of probiotics and the duration of probiotic administration. In addition, we evaluated the possible reasons of heterogeneity by sensitivity analysis after removing one high‐risk bias or two unclear risk biases to evaluate the stability of results.

## 3 RESULTS

### 3.1 Study inclusion and characteristics

**FIGURE 1:**
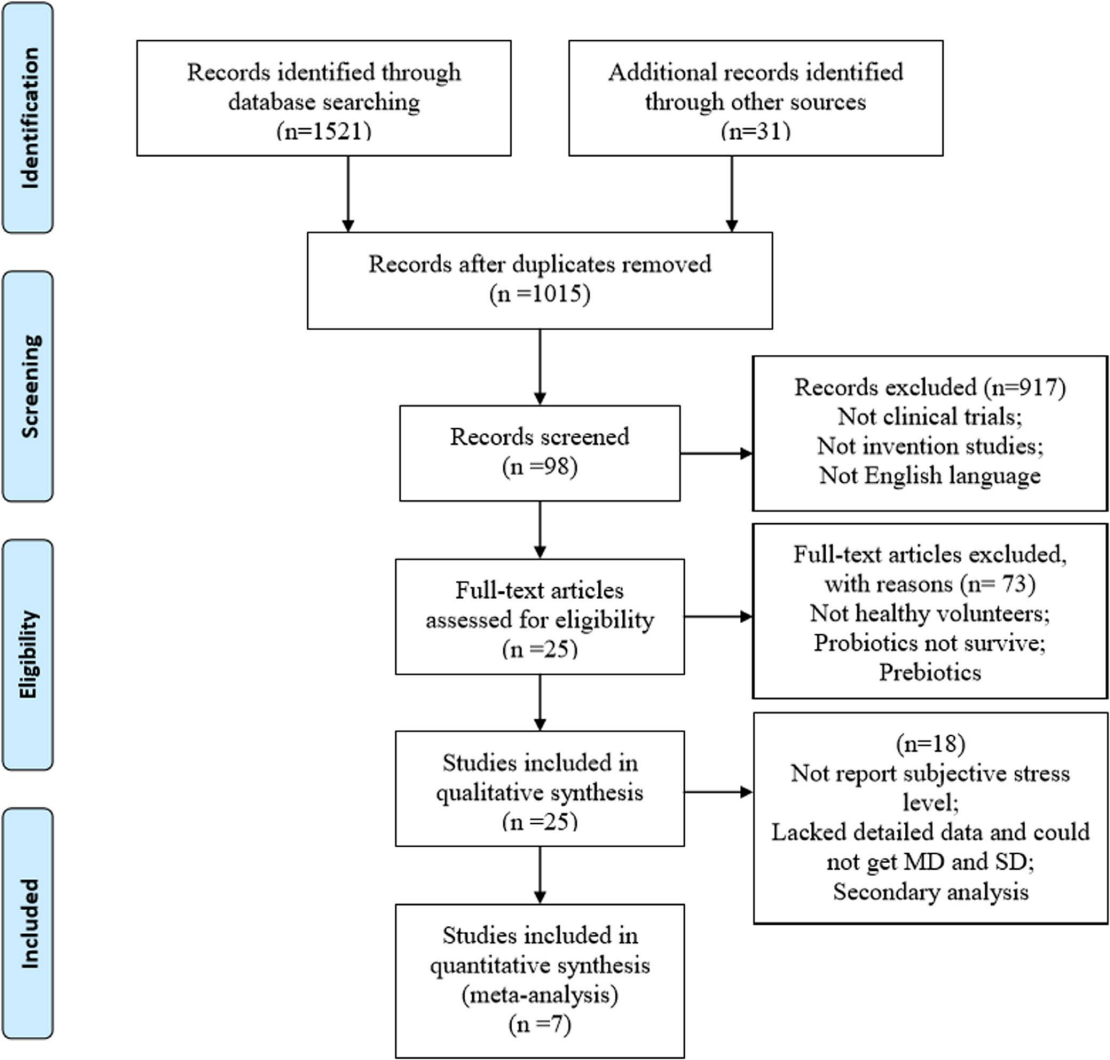
An adapted PRISMA Flow Diagram

An adapted PRISMA Flow Diagram of the article search and trial selection process was shown in Figure 1. A total of 1,552 potentially relevant records were selected. After duplicates removed, 917 records were excluded. We screened the title and abstract and then assessed the full texts. Finally, 25 studies met the inclusion criteria. However, outcome indicators for nine studies did not include subjective stress level. Eight studies lacked detailed description or could not get MD and SD. One study was described as secondary analysis of RCT. Thus, seven studies were included in quantitative synthesis. The characteristics of 18 major excluded studies for meta‐analysis are provided in Table S1.

The characteristics of each eligible study were shown in Table 1. There were no significant statistical differences in the outcome indicators we focus on in seven studies at baseline (Chung et al., 2014; Langkamp‐Henken et al., 2015; Lew et al., 2018; Makino et al., 2018; Messaoudi et al., 2011; Ostlund‐Lagerstrom et al., 2016; Sanchez et al., 2017). Östlund‐Lagerström 2016 and Lew 2018 reported the results in baseline changes. The probiotic administration of Östlund‐Lagerström 2016 was divided into three groups: Lactobacillus helveticus R0052, *Bifidobacterium bifidum* R0071, and *Bifidobacterium longum* ssp. Infantis R0033; In Chung 2014, the probiotics were divided into three doses of 500 mg, 1,000 mg, and 2,000 mg. According to the calculation methods in the Cochrane handbook, we merged the relevant studies (Higgins & Green, 2011). Messaoudi 2011 measured the subthreshold anxiety/depression level of the subjects twice using the HSCL‐90 and HADS scales, and we included the outcomes of both scale measurements into the meta‐analysis. In addition, the Messaoudi 2011 reported median, first quartile, third quartile, and sample size, and we calculated the mean and *SD* according to the method by Wan et al (Wan, Wang, Liu, & Tong, 2014).

**TABLE 1:** Characteristics of included RCTs for meta‐analysis

Study, year, location	Volunteer, treated/total	Intervention (strain), daily dose	Comparison	Duration	Main outcomes, measures
Messaoudi, 2011, France	Healthy participants, 26/55	Probio‐Stick (*L. helveticus R0052* and *B. longum R0175*), 3 × 10^9^ CFU	Placebo stick of identical taste and appearance, containing xylitol, maltodextrin, plum flavor, and malic acid	30 days	1. subjective stress level: PSS; 2. stress‐related subthreshold anxiety and depression: HSCL‐90, HADS; depression: HSCL‐90, HADS; 3. cortisol level in urine: Elisa
Chung, 2014, Korea	Healthy elderly participants, 26/36	Tablet (*L. helveticus IDCC3801*), 500, 1,000, or 2000 mg of LHFM	Placebo tablets of identical color, shape, and size	12 weeks	1. subjective stress level: PSS; 2. stress‐related subthreshold anxiety and depression: GDS‐SF
Langkamp‐Henken, 2015, United States	healthy, full‐time undergraduate adult students who reported at least one cold in the past year, 147/581	Capsule (*Lactobacillus helveticus R0052*, *Bifidobacterium longum* ssp. *infantis R0033*, *Bifidobacterium bifidum R0071*), 3 × 10^9^ CFU	A placebo capsule of similar appearance containing an off‐white powder consisting of 3% magnesium stearate and 97% potato starch	6 weeks	1. subjective stress level: a Web‐based survey tool (Qualtrics)
Östlund‐Lagerström, 2016, Sweden	free‐living older adults,125/249	Stick‐pack (freeze‐dried *L. reuteri DSM 17938*), 2 × 10^8^ CFU	The placebo product consisted of maltodextrin with the same appearance, color and taste as the active study product, identically packaged and stored	12 weeks	1. subjective stress level: PSS; 2. stress‐related subthreshold anxiety and depression: anxiety: HADS; depression: HADS; 3. possible adverse reactions
Sanchez, 2017, Canada	Participants with obesity, 62/125	Capsule (*Lactobacillus rhamnosu*s), 3.24 × 10^8^ CFU	The placebo capsules were the same color and size and contained 250 mg of maltodextrin and 3 mg of magnesium stearate	24 weeks	1. subjective stress level: PSS; 2. stress‐related subthreshold anxiety and depression: anxiety: STAI; depression: BDI
Makino, 2018, Japan	Healthy people suffering from summer heat fatigue, 25/49	Yogurt (*L. bulgaricus OLL1073R‐1* and *Streptococcus thermophilus OLS305*9), 100 ml	Yogurt fermented with a placebo	12 weeks	1. subjective stress level: VAS
Lew, 2018, Malaysia	Healthy adult participants at moderate stress level, 52/103	Powder (*L. plantarum P8*), 2 × 10^10^ CFU	A pale yellow powder with the same appearance, taste, and packaging	12 weeks	1. subjective stress level: PSS; 2. cortisol level in plasma: ELISA

Abbreviations: BDI, Beck Depression Inventory; ELISA, enzyme‐linked immunosorbent assay; GDS‐SF, Geriatric Depression Scale‐Short Form; HADS‐A, Hospital Anxiety and Depression Scale; HSCL‐90, Hopkins Symptom Checklist‐90; PSS, Perceived Stress Scale; STAI, State‐Trait Anxiety Inventory; VAS, Visual Analog Scales.

### 3.2 Bias assessment

**FIGURE 2:**
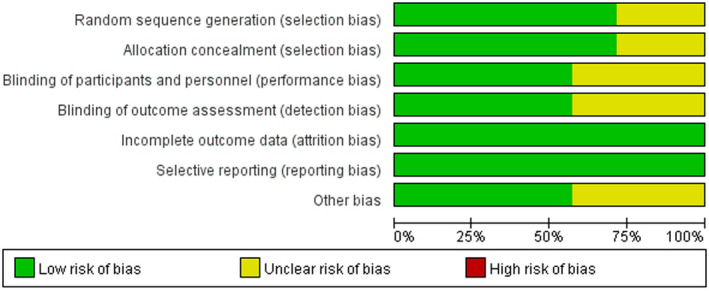
Risk of bias for each included study

**FIGURE 3:**
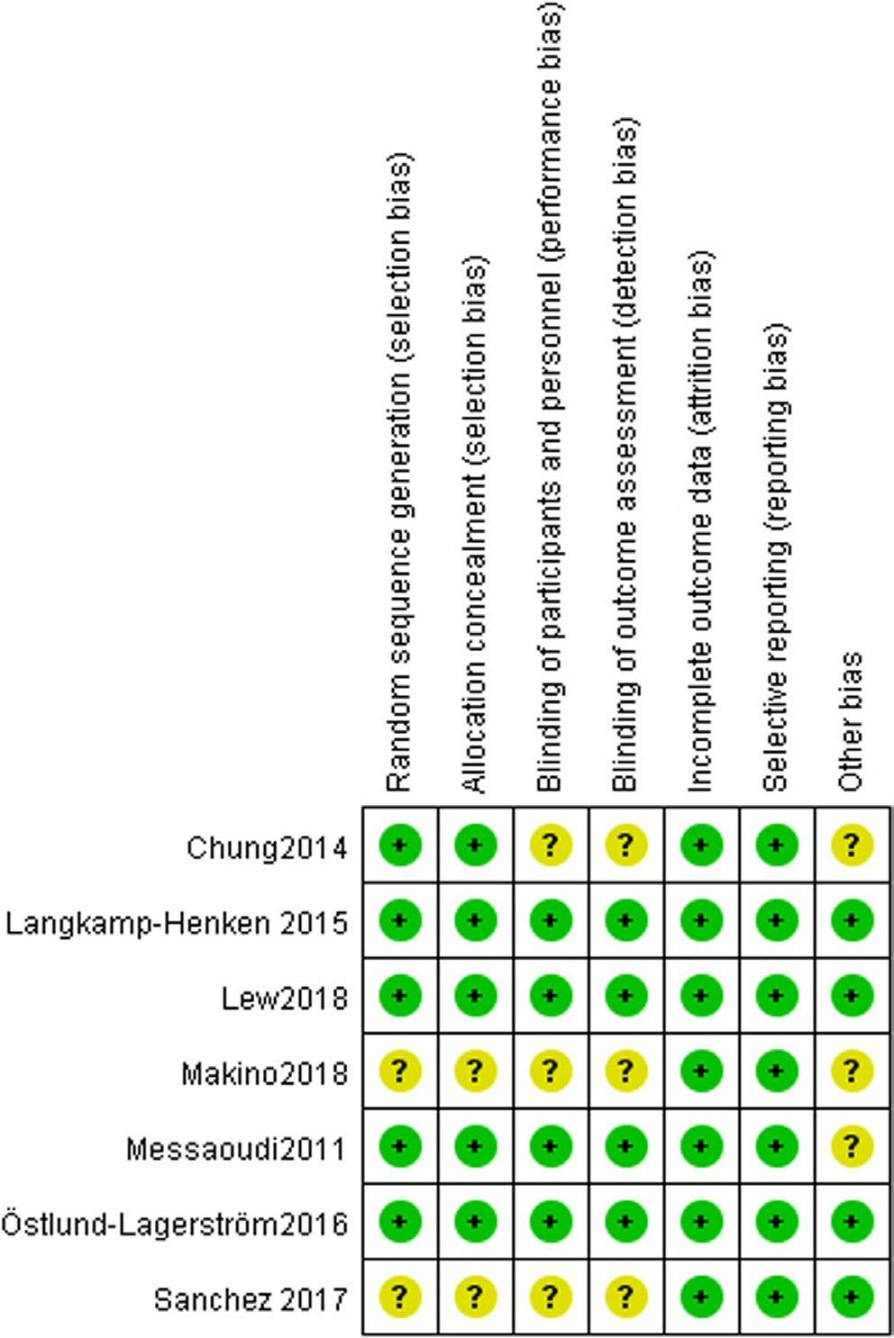
The percent risk of bias for each included study

Risk of bias for each study included in meta‐analysis was shown in Figures 2 and 3 compared the percent risk of bias, indicating that the risk of bias was at a moderate level. Moreover, it is necessary to note that Makino 2018 was conducted by researchers at Meiji Co., Ltd. of Japan, which involved R test products produced by R&D division in Meiji Co., Ltd. In the report, they stated that there was no conflict of interest (Makino et al., 2018), but we have reservations about it and determined other bias as unclear risk.

### 3.3 Outcomes

#### 3.3.1 Subjective stress level

**FIGURE 4:**
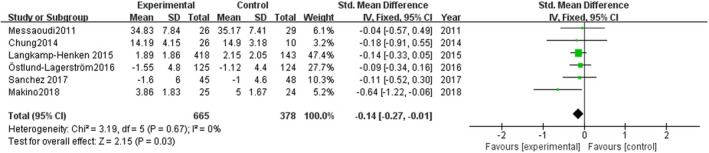
Effect of probiotics on subjective stress levels

As shown in Figure 4, the meta‐analysis comparing the experimental group and control group showed SMD = −0.14, 95% CI: −0.27 to −0.01, *p* = .03. Heterogeneity test in six studies might not be important.

**FIGURE 5:**
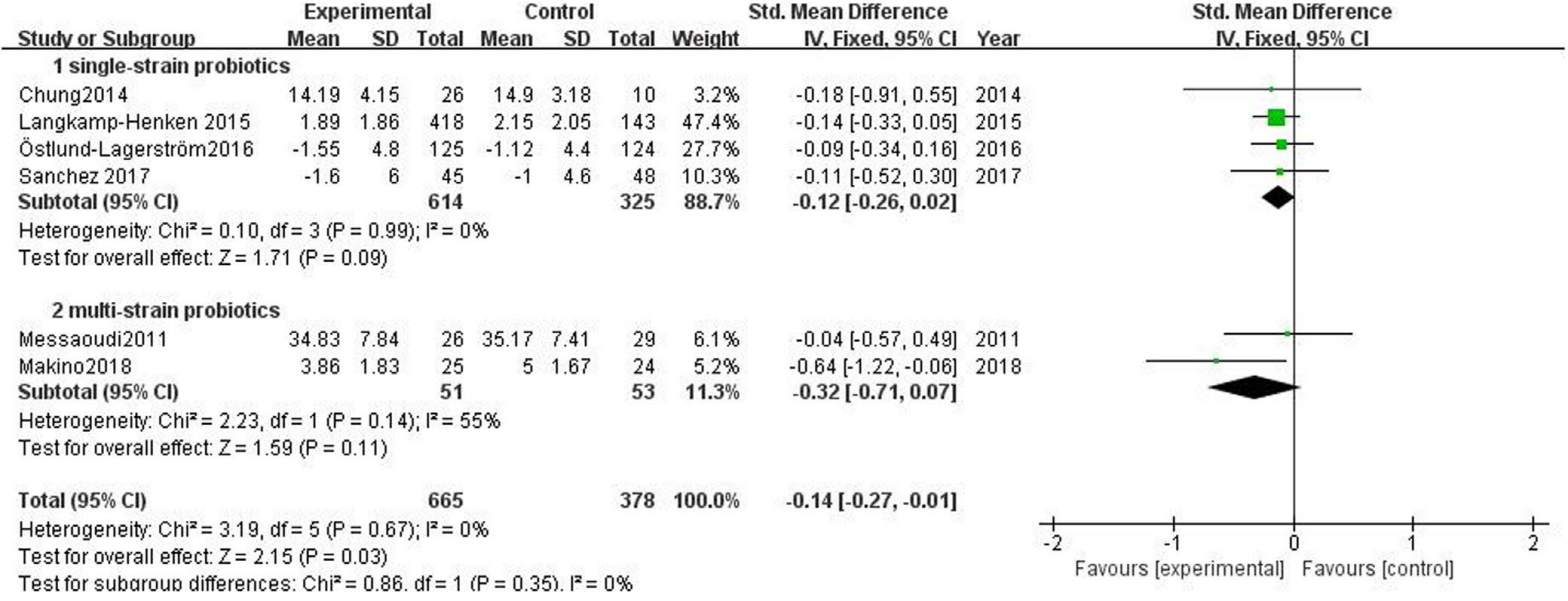
Effect of single‐strain and multi‐strain probiotics on subjective stress levels

**FIGURE 6:**
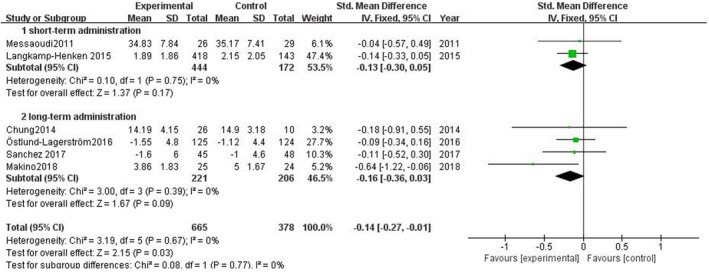
Effect of short‐term and long‐term probiotic administration on subjective stress levels

Subgroup analyses focused on the type/duration of probiotics to observe the effect of probiotics on subjective stress level (Figure 5, Figure 6). Single‐strain probiotics were used as invention in four studies, SMD = −0.12 (95% CI: −0.26 to 0.02), *p* = .09; in other two studies, multi‐strain probiotics were used, SMD = −0.32 (95% CI: −0.30 to 0.05), *p* = .11. The volunteers in two studies were arranged to short‐term administration, SMD = −0.13 (95% CI: −0.30 to 0.05), *p* = .17. The volunteers in other four studies were arranged to long‐term administration, SMD = −0.16 (95% CI: −0.36 to 0.03), *p* = .09.

#### 3.3.2 Stress‐related subthreshold anxiety/depression level

**FIGURE 7:**
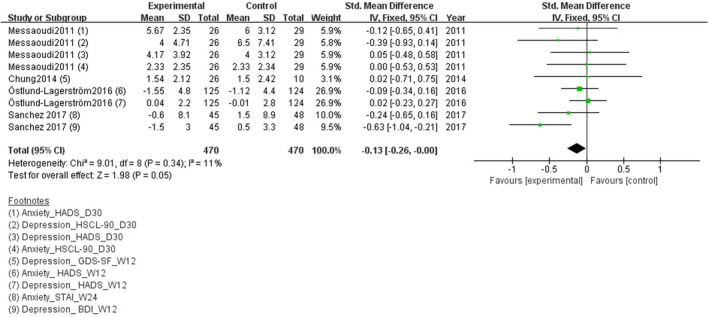
Effect of probiotics on stress‐related subthreshold anxiety/depression level

According to Figure 7, the meta‐analysis of four studies showed no significant changes in stress‐related subthreshold anxiety/depression comparing the experimental group and the control group, SMD = −0.13 (95% CI: −0.26 to 0.00), *p* = .05.

**FIGURE 8:**
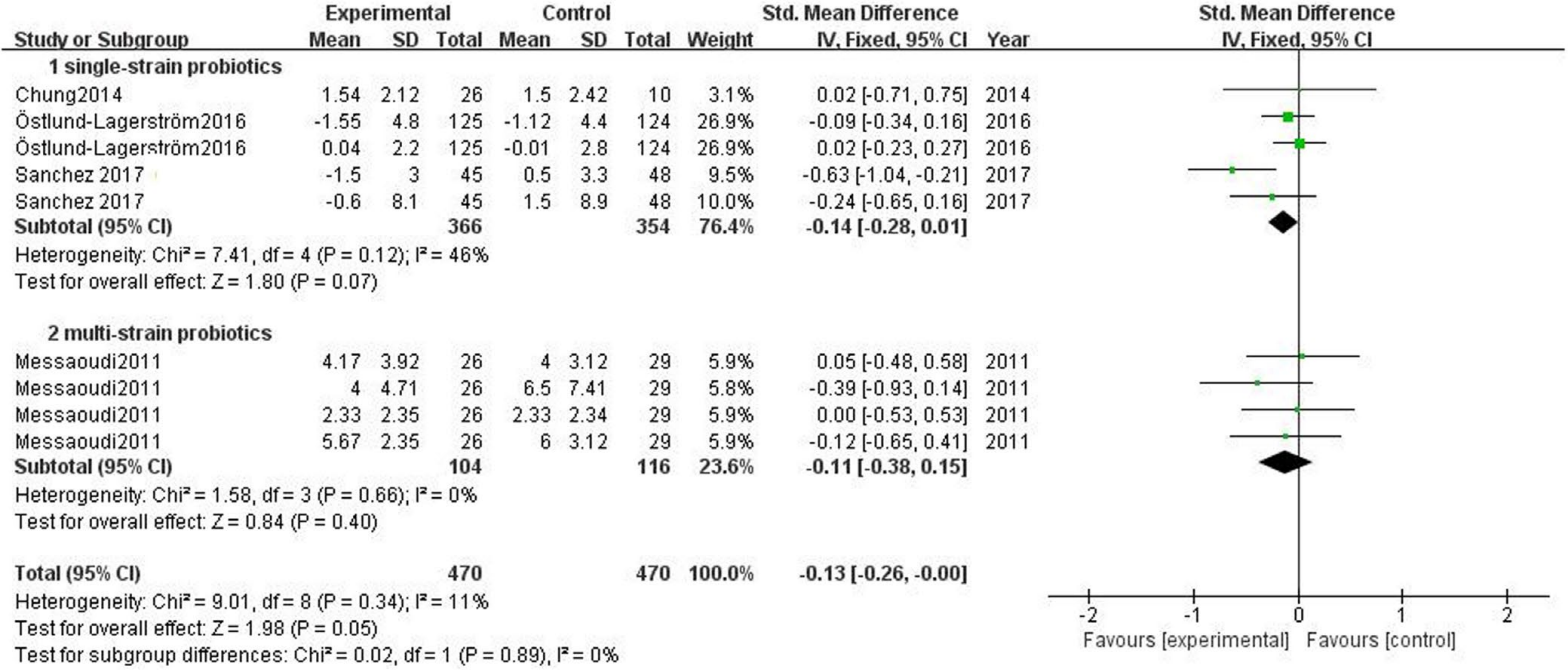
Effect of single‐strain and multi‐strain probiotics on stress‐related subthreshold anxiety/depression level

**FIGURE 9:**
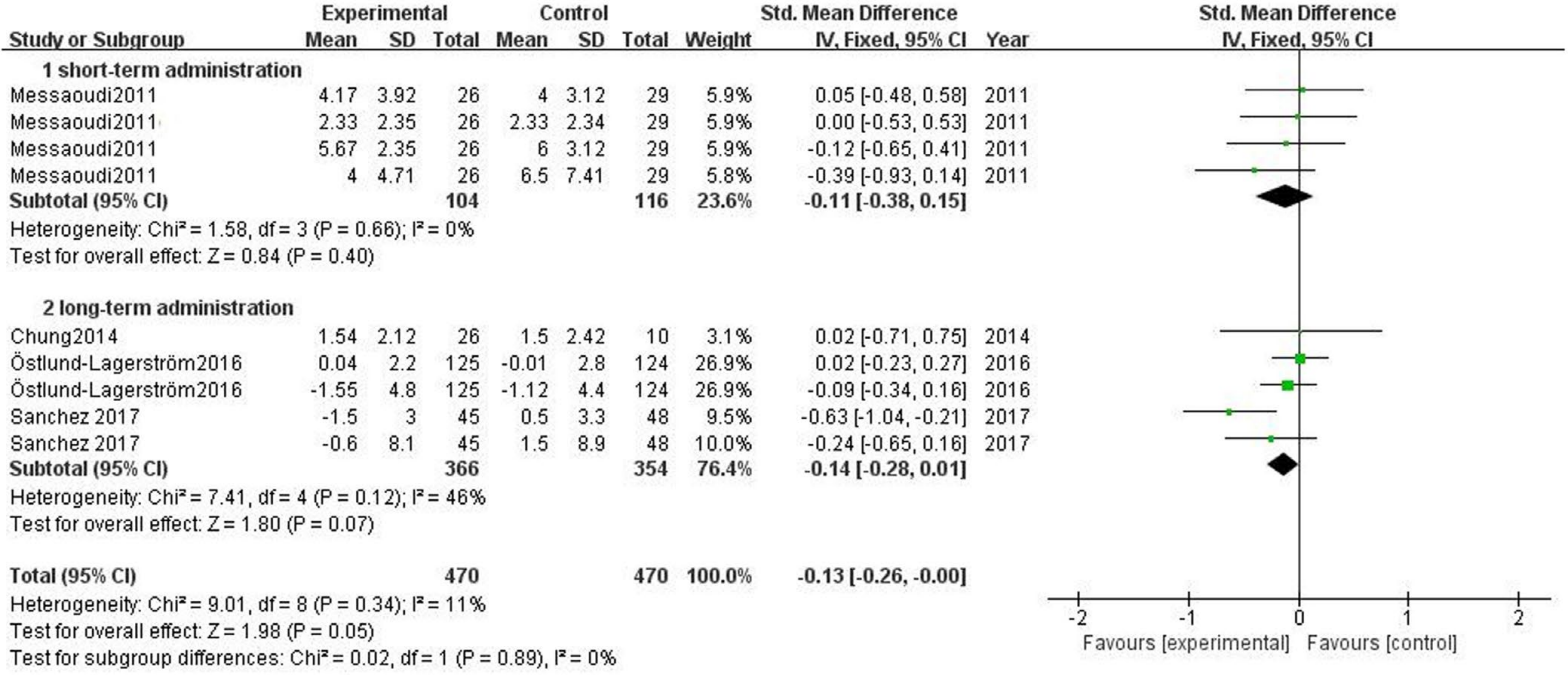
Effect of short‐term and long‐term probiotic administration on stress‐related subthreshold anxiety/depression level

Subgroup analyses showed that in three studies of single‐strain probiotics, SMD = −0.14 (95% CI: −0.28 to 0.01), *p* = .07; in one study of multi‐strain probiotics, SMD = −0.11 (95% CI: −0.38 to 0.15), *p* = .40. The volunteers in one study were arranged to short‐term administration, SMD = −0.11, (95% CI: −0.38 to 0.15), *p* = .40. The volunteers in four studies were arranged to long‐term administration, SMD = −0.14 (95% CI: −0.28 to 0.01), *p* = .07 (see Figures 8, 9 for details).

#### 3.3.3 Cortisol level

**FIGURE 10:**
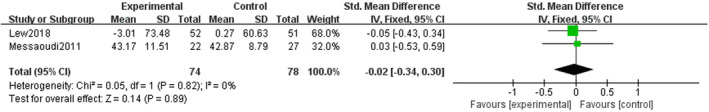
Effect of probiotics on the cortisol level

According to Figure 10, the quantitative synthesis of two studies showed SMD = −0.02 (95% CI: −0.34 to 0.30), *p* = .89. Due to limited number of studies of probiotic administration on the influence of cortisol level, we did not conduct a subgroup analysis.

#### 3.3.4 Adverse reactions that may be associated with probiotic administration

Only one RCT (Ostlund‐Lagerstrom et al., 2016) reported adverse reactions that may be associated with probiotics. In the report, there was no detailed description about adverse reaction, and we attempted to contact the first author and correspondent author of the study by email but failed, so no further analysis was made.

### 3.4 Reporting bias and sensitivity analysis

According to the protocol (Zhang et al., 2019), when the number of studies included is more than 10, we will evaluate the possible reporting bias through the funnel plot, but this study cannot satisfy this condition on a single outcome indicator. According to the comprehensiveness of the retrieval, we are confident that there are not many missed retrieval, but there is no guarantee that there will be a possibility that the relevant negative results are not published. We hope more qualified researches in the future can be used to conduct funnel plots and related Egger's and Begg's tests, and to determine reporting bias quantitatively and qualitatively.

### 3.5 Sensitivity analysis

We found remarkable changes in SMD and heterogeneity after removing two or more unknown bias risk studies item by item, which indicated certain unstable results. As shown in Figures S1 and S2, the effect of probiotics on subjective stress level changed greatly and made the effect of probiotics on subjective stress level less significant when Makino 2018 was removed, and removal of the data of stress‐related subthreshold anxiety/depression level measured by BDI in Sanchez 2017 imposed a great influence on results.

## 4 DISCUSSION

Psychological stress is common in everyday life, and most of the stress experience is accompanied by physiological and psychological responses (Campbell & Ehlert, 2012; McEwen & Seeman, 1999). The impact of stress on individuals is a process of gradual accumulation, and each additional stress experience increases the overall adaptation burden and even undermines the health, which has gradually become the main problem in daily life (Cohen, Gianaros & Manuck, 2016).

Recent studies on microbiome have found that the intestinal microbes and the central nervous system are closely linked through neural pathways, metabolic pathways, and immune pathways (Rieder, Wisniewski, Alderman, & Campbell, 2017). A variety of stimulating factors may produce a known or unknown effect on the function status of the central nervous system and the microbiome through this “bottom‐up” or “top‐down” path (Bienenstock, Kunze, & Forsythe, 2015). The same is true of stress. It has been confirmed that the microbiome is associated with the development of stress‐related diseases such as mental disorders. Experiments in animals found that it would cause depression‐like/anxiety‐like behavior when destroying the microbiome (Breit & Chester, 2016; Karrenbauer et al., 2011), and clinical studies conducted by Jiang et al. have found that compared with healthy control group, changes in the intestinal flora of patients with depression, which also support this view (Jiang et al., 2015). Therefore, the researchers speculate that it is possible to reduce the impacts of stressful events on individuals by regulating intestinal microbes.

A large number of clinical trials based on microbiome‐based innovative therapies (such as probiotics, prebiotics, and fecal transplants) have been carried out for stress‐related diseases at present; thus, we conducted this research with a clear focus and address question that whether probiotic management is effective to stress. There are marked differences in the methods and results between our study and the studies by Romin 2015 (Romijn & Rucklidge, 2015) and Mckean (Mckean, Naug, Nikbakht, Amiet, & Colson, 2017). Romin & Rucklidge conducted study earlier and had no restrictions on the health condition of the subjects, and only included one randomized controlled trial in healthy volunteers (Messaoudi et al., 2011), and the results did not show that probiotics could reduce subjective stress level and cortisol level. Mckean et al. comprehensively analyzed the overall effects of probiotics on subjective stress level, anxiety, and depression.

Whether the stress affects individuals mainly depends on the individual's subjective interpretation of stress events. The higher the subjective stress level, the greater the psychological stress the individual suffers (Kuiper, Olinger & Lyons, ; Cohen, Kamarck, & Mermelstein, 1983). As is well‐known, stress is closely related to mental health (Marin et al., 2011). Etiological studies showed that excessive stress responses would cause psychological discomfort, such as anxiety (Schneiderman et al., 2005), depression (Tafet & Nemeroff, 2016), and even mental illness (Gehrman et al., 2015; Slavich & Irwin, 2014). Thus, efficacy of probiotics on stress‐related anxiety/depression was selected as another main outcome measure with the consideration of feasibility. Different from previous studies, we only included studies that reported subjective stress level to ensure a correlation between anxiety/depression and stress, which is also an important reason for different conclusions of our study, and it is also a feature of our study.

Seven RCTs were included to explore the relationship between the duration of probiotic administration and its psychological effects. Efficacy is usually evaluated after 8‐week intervention in the clinical practice of mental disorders such as anxiety and depression. Based on this, we conducted the subgroup analysis based on long‐term administration (≥8 weeks) and short‐term administration (<8 weeks). In addition, probiotics currently on the market include single‐strain and multi‐strain probiotic products (Fijan, Sulc, & Steyer, 2018; Korada et al., 2018). Although the current research on the two types of products does not have definite conclusions, it is generally believed that different probiotic strains will have multiple levels of interaction, which can share different metabolites and affect the use of different metabolites, and also produce more bioactive substances. Therefore, we performed subgroup analysis according to different probiotic product categories (Chapman, Gibson & Rowland, 2012). Results have shown that probiotic administration can generally reduce the subjective stress level of healthy subjects (*p* = .03) and may improve their stress‐related subthreshold anxiety/depression level (*p* = .05), thus showing potential “psychobiotics” properties of probiotics. However, affected by factors such as the number and sample size of included studies, the exploratory subgroup analysis did not show a positive result (*p* > .05), so this phenomenon is also reasonable and understandable.

Although the effects of single‐strain probiotics and multi‐strain probiotics or short‐term and long‐term probiotic administration on subjective stress level and subthreshold anxiety/depression level are not statistically significant, probiotics can decrease the subject's subjective stress level (*p* = .03) and possibly improve subthreshold anxiety/depression level (*p* = .05), showing potential “psychobiotics” properties of probiotics.

Based on its extensive evidence of The Trier Social Stress Test, cortisol is also known as the “emergency hormone” (Allen, Kennedy, Cryan, Dinan, & Clarke, 2014), and we deemed cortisol level as an indicator of the effect of probiotic on physiological stress level. Nevertheless, only involved 152 healthy subjects from two studies, and we cannot come up with convincing conclusion.

Only one study in our study reported possible adverse reactions associated with probiotics, but there was no detailed description of adverse reactions. Thus, there is not enough support to draw conclusions about adverse effects, which should be paid great attention in clinicians.

There were still some limitations in this study. First, same as the previous systematic reviews and meta‐analyses, the number of included studies is limited. Some small sample size studies were inevitably included during our analysis, which would affect the credibility of the results; second, different psychological scales are also a possible reason for heterogeneity; third, different restrictions on dietary structures in subjects in included studies will impose an unknown influence on the outcomes; fourth, included studies were with different objectives, conducted in different countries, and subjects in different genetic backgrounds, ages, and lifestyles, which may affect the results; last but not least, the more critical point from the previous studies is the limitation of current knowledge, especially given the marked interstudy variability in terms of probiotic strain. Not all probiotics have the same effect on stress. It is also necessary to match the appropriate probiotic strain or mixture to the needs of the patients. Every effort should be made to report specific probiotic strains or mixture of strains when analyzing the efficacy and safety of probiotics in further studies.

## 5 CONCLUSIONS

This systematic review and meta‐analysis showed that probiotic administration could reduced subjective stress level in healthy volunteers and may relieve stress‐related subthreshold anxiety/depression level, but the effect on cortisol was not significant. In addition, adverse reactions were left undescribed; then, there is not enough support to draw conclusions about adverse effects. However, since the results of meta‐analysis are still unstable, there is a need for more reliable clinical evidence support and a deeper understanding of the strain specificity of probiotics before probiotics can be used to relieve stress.

## CONFLICT OF INTERESTS

The authors declare no conflict of interest.

## AUTHOR CONTRIBUTION

**Ning Zhang** and **Shuangqing Zhai** involved in conceptualization. **Ning Zhang**, **Yanan Zhang,** and **Menglin Li** involved in data curation. **Ning Zhang** and **Yanan Zhang** involved in formal analysis. **Zhenzhu Liu** and **Chongcheng Xi involved** in investigation. **Xunying Huang**, **Jintao Liu**, **Junwei Huang**, **Dong Tian**, and **Jie Mu** involved in Software. **Shuangqing Zhai** involved in funding acquisition. **Xing Liao** and **Weiguang Wang** involved in methodology. **Ning Zhang**, **Xing Liao,** and **Shuangqing Zhai** involved in project administration. **Ning Zhang** involved in writing–original draft. **Xing Liao** and **Shuangqing Zhai** involved in writing–review and editing.

## ETHICAL APPROVAL

All analyses were based on previous published studies; thus, no ethical approval and patient consent are required.

## Supporting information

Supplementary Material

## Data Availability

All data generated or analyzed for supporting the findings of this study are available from the corresponding author upon reasonable request.
