# Supplementary material for: Efficacy of probiotics on stress in healthy volunteers: A systematic review and meta‐analysis based on randomized controlled trials
Source: Brain Behav. 2020 Jul 14;10(9):e01699. doi: 10.1002/brb3.1699 (PMC7507034; doi:10.1002/brb3.1699)
Supplement: Supplementary file 1 — Supplementary Material [file BRB3-10-e01699-s001.docx]

| **Table S1. Characteristics of major excluded studies for meta-analysis** | | | | | |
| --- | --- | --- | --- | --- | --- |
| **Study** | **Volunteers, Sample size, Type of Study** | **Intervention, Duration** | **Comparison, Duration** | **Outcomes** | **Reasons for exclusion** |
| Mohammadi 2015 | Petrochemical workers without major health problems, 20/70, randomized controlled trial | Probiotic yogurt & placebo capsule or probiotic capsule & conventional yogurt, 6 weeks | Conventional yogurt & placebo capsule, 6 weeks | 1. Psychological parameters: GHQ, DASS; 2. Kynurenine, Tryptophan, ACTH, etc. | Not report subjective stress level: unable to get the score of stress subscale in DASS |
| Möller 2017 | Healthy young students, 57/105, randomized controlled trial | Probiotic capsule, 6 weeks | Placebo capsule, 6 weeks | 1. Cardiovascular activities :SBP, DBP, and HR; 2. Psychological reactions: acute psychological stress task, etc. | Not report subjective stress level: unable to get the score of stress in acute psychological stress task |
| Kelly 2017 | Healthy male volunteers, 29/58, a repeated measures cross-over design | Probiotic capsule, 4 weeks | Placebo capsule, 4 weeks | 1. Tests from the CANTAB Battery; 2. Acute Stress Procedure; 3. Neurocognitive assessment; 4. Resting EEG; 5. Biomarkers: Cortiso, Cytokine, and TLR-4 cytokine, etc. | Could not get MD and SD: the results are shown in the figure |
| Allen 2016 | Healthy male volunteers, 22/44, a repeated measures design | Probiotic stick, 4 weeks | Placebo stick, 4 weeks | 1. Self-report; 2. Cognitive tasks; 3. Rapid visual information processing; 4. Emotion Recognition Task, etc. | Could not get MD and SD |
| Diop 2008 | Volunteers with symptoms of stress, 31/64, randomized controlled trial | Probiotic Stick, 3 weeks | Placebo stick, 3 weeks | 1. Stress-induced physical and psychological symptoms; 2. Stress-induced gastrointestinal symptoms; 3. adverse reactions | Not report subjective stress level |
| Kato-Kataoka 2016 | Healthy medical students, 24/47, randomized controlled trial | *LcS*-fermented milk, 8 weeks | Placebo milk, 8 weeks | 1. Psychological parameters; 2. Biomarkers: Salivary cortisol and secretory immunoglobulin A levels, Plasma L-tryptophan and L-ynurenine levels, and Faecal serotonin level; 3. Physical symptoms, etc. | Not report subjective stress level |
| Kato-Kataoka 2015 | Healthy medical students, 23/47, randomized controlled trial | *L*.casei strain Shirota-fermented milk, 8 weeks | Placebo milk, 8 weeks | 1.Psychological parameters: HPI, GHQ-28, VAS, and STAI; 2. Biomarks: Salivary cortisol and alpha-amylase, NK cells in blood, DNA microarray, Ampli consequencing of the gut microbiota targeting the 16S rRNA gene, etc. | Could not get MD and SD: the results are shown in the figure |
| Takada 2016 | Healthy medical students, 70/140, randomized controlled trial | *LcS*-fermented milk, 8 weeks | Non‐fermented placebo milk, 8 weeks | 1. Changes in psychological parameters; 2. Changes in salivary cortisol levels; 3. Changes in incidence rate of physical symptoms, etc. | Not report subjective stress level |
| Takada 2017 | Healthy medical students, 48/94, randomized controlled trial | *LcS*-fermented milk, 8 weeks | Non-fermented placebo milk, 8 weeks | 1. Psychological parameters: GHQ, NEO-FFI, STAI, and PSQI; 2. Overnight single-channel electroencephalography; 3. The Oguri-Shirakawa-Azumi (OSA) sleep inventory scores of subjective sleep quality, etc. | Not report subjective stress level |
| Marcos 2004 | Healthy students , 62/114, randomized controlled trial | *LcS*-fermented milk, 6 weeks | Placebo milk not containing LcS, 6 weeks | 1. Psychological parameters: STAI; 2. Biomarks: Cortisol, Cytokines, and Immunoglobulins, etc. | Not report subjective stress level |
| Culpepper 2016 | Healthy adult students, 434/581, randomized controlled trial | Probiotic capsule, 6 weeks | Placebo capsule, 6 weeks | 1. Psychological parameters: self-reported stress levels; 2. Gastrointestinal Symptom; 3. Diarrhoea syndrome, etc. | Secondary analysis |
| Smith 2013 | College students, 114/231, randomized controlled trial | Probiotic powder, 12 weeks | Placebo powder, 12 weeks | 1. Health-related quality of life; 2. Missed school and work days, etc. | Not report subjective stress level |
| Gomi 2018 | Healthy adults, 50/100, randomized controlled trial | *YIT10347*-fermented milk, 4 weeks | Placebo fermented milk, 4 weeks | 1. Gastrointestinal Symptom; 2. Subjective psychological symptoms; 3. Subjective quality of life; 4. Biomarks: Salivary Cortisol, etc. | Not report subjective stress level |
| Talbott 2019 | Healthy subjects, 21/32, randomized controlled trial | Supplement containing probiotic, prebiotic, phytobiotic plant extracts and nutrients, 1 month | Placebo, 1 month | 1. Microbiome Assessment; 2. Mood Assessment: POMS, etc. | Not report subjective stress level |
| Andersson 2016 | Young adults under examination stress, 21/41, randomized controlled trial | Probiotic capsule, 2 weeks | Placeebo capsule, 2 weeks | 1. Psychological Assessment: perceived stress; 2. Levels of Cortisol and IgA in Saliva, etc. | Could not get MD and SD |
| Wang 2019 | Healthy adults, 20/40, randomized controlled trial | Probiotic sachet, 4 weeks | Placebo sachet, 4 weeks | 1. Psychological parameters: CBG questionnaires; 2. MEG recording, etc. | Not report subjective stress level |
| Papalini 2018 | Healthy participants, 29/58, randomized controlled trial | Probiotic powder, 4 weeks | Placebo powder, 4 weeks | 1. Psychological parameters: BDI, and LEIDS-r; 2. fMRI; 3. the socially evaluated cold pressor test, etc. | Could not get MD and SD: the results are shown in the figure |
| Carbuhn 2018 | Collegiate female swimmers, 10/20, randomized controlled trial | Probiotic capsule, 6 weeks | Placebo capsule, 6 weeks | 1. General Performance Testing; 2. Inflammatory/Immune Markers; 3.Cognitive Stress-Recovery Assessment: General Stress, General Recovery,Sport Stress, and Sport Recovery, etc. | Not report subjective stress level: unable to get the total score of General Stress |

Notes: ACTH, adreno cortico tropic hormone; SBP, systolic blood pressure; DBP, diastolic blood pressure; HR, heart rate; EEG, electroencephalography; NEO-FFI, NEO Five-Factor Inventory; PSQI, Pittsburgh sleep quality index; HPI, Hogan personality inventory; MEG, magnetoencephalogram; LEIDS-r, Leiden Index of Depression Sensitivity-Revised; fMRI, functional magnetic resonance imaging.


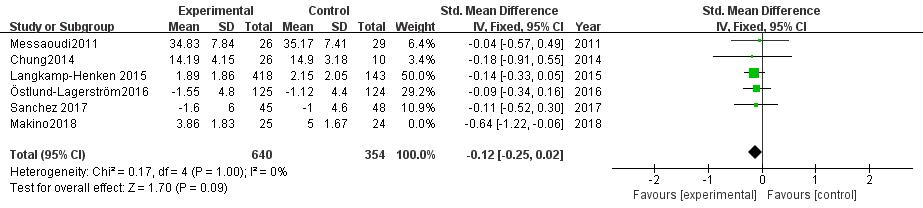


Figure S1. sensitivity analysis 1


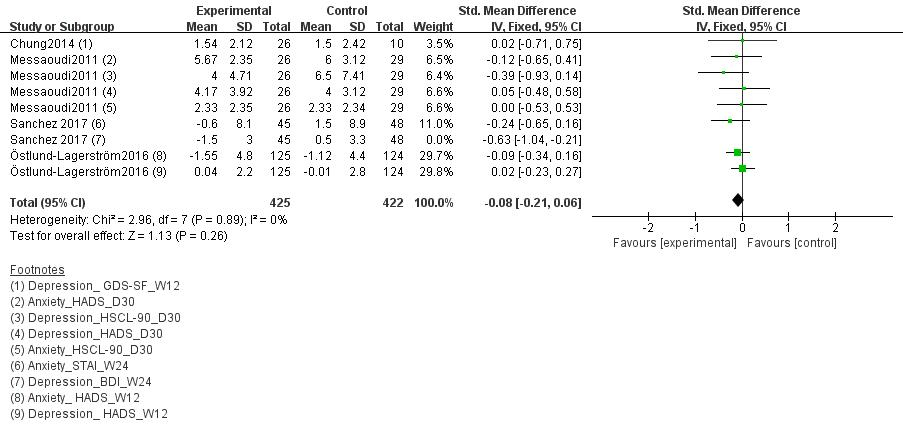


Figure S2. sensitivity analysis 2
